# What is the lifetime risk of developing cancer?: the effect of adjusting for multiple primaries

**DOI:** 10.1038/bjc.2011.250

**Published:** 2011-07-19

**Authors:** P D Sasieni, J Shelton, N Ormiston-Smith, C S Thomson, P B Silcocks

**Affiliations:** 1 Centre for Cancer Prevention, Wolfson Institute of Preventive Medicine, Bart's and The London School of Medicine, Queen Mary University of London, London EC1M 6BQ, UK; 2 National Cancer Intelligence Network, 18th Floor, Portland House, Bressenden Place, London SW1E 5RS, UK; 3 Cancer Research UK, Statistical Information Team, Angel Building, 407 John Street, London EC1V 4AD, UK; 4 Cancer Research UK Liverpool Cancer Trials Unit, University of Liverpool, Block C Waterhouse Building 1-3 Brownlow Street, Liverpool L69 3GL, UK

**Keywords:** cancer incidence, cancer-free survival, lifetime risk

## Abstract

**Background:**

The ‘lifetime risk’ of cancer is generally estimated by combining current incidence rates with current all-cause mortality (‘current probability’ method) rather than by describing the experience of a birth cohort. As individuals may get more than one type of cancer, what is generally estimated is the average (mean) number of cancers over a lifetime. This is not the same as the probability of getting cancer.

**Methods:**

We describe a method for estimating lifetime risk that corrects for the inclusion of multiple primary cancers in the incidence rates routinely published by cancer registries. The new method applies cancer incidence rates to the estimated probability of being alive without a previous cancer. The new method is illustrated using data from the Scottish Cancer Registry and is compared with ‘gold-standard’ estimates that use (unpublished) data on first primaries.

**Results:**

The effect of this correction is to make the estimated ‘lifetime risk’ smaller. The new estimates are extremely similar to those obtained using incidence based on first primaries. The usual ‘current probability’ method considerably overestimates the lifetime risk of all cancers combined, although the correction for any single cancer site is minimal.

**Conclusion:**

Estimation of the lifetime risk of cancer should either be based on first primaries or should use the new method.

Within limits, cancer registries register details on all primary tumours that arise in individuals in their catchments area, and routinely published incidence data include more than one cancer for some individuals (those with a second or multiple primaries). To quantify the burden of cancer in a population, traditionally, epidemiologists present and compare age-standardised incidence rates, which is an approach still taken by the International Agency for Research on Cancer (IARC) in its Cancer in Five Continents series (Parkin *et al*, 2010), but these have little intuitive appeal. By contrast, the lay media likes to quote ‘lifetime risk’ (e.g., ‘1 in 3 of us will get cancer at some point in our lives’), but currently the calculation of this may not correspond to what one intuitively understands by the phrase; it is generally not comparable between populations, or over time.

Here we describe briefly the various existing methods which could be used to give an estimate of lifetime risk, and why some of them are not advisable. We also highlight the distinction between the probability of getting cancer over a lifetime (the true lifetime risk) and the mean number of cancers per lifetime (which is currently often what is estimated by the reported ‘lifetime risk’). Finally, we propose a method for estimating the true lifetime risk from routine national statistics, allowing for both competing risks to be taken into account, and for avoiding second primaries in the same individual being treated as if the cancers were in two different individuals. The effect of this adjustment is to reduce the estimated lifetime risk of cancer: the resulting estimate is close to that obtained when calculations can be performed on data including only first primaries in individuals (which we propose is the ‘gold standard’ for calculating lifetime risk).

**Summary of existing techniques measuring cancer in the population, and the risk of developing cancer**

*Crude and age-standardised incidence rates* The simplest method, which summarises the occurrence of disease in a population is the crude incidence rate. As the incidence of cancer varies hugely with age, this measure suffers two main disadvantages: it has no ‘everyday’ interpretation; and direct comparison between populations is likely to be misleading because of different age structures. The effect of age can be controlled by the process of direct age standardisation, but this generally gives a figure that is even less intuitively interpretable. Neither of these methods provides estimates of the lifetime risk of developing cancer.

*Cumulative rate* Day, 1987 proposed a different method of age-standardising incidence rates called the cumulative rate, defined as: 
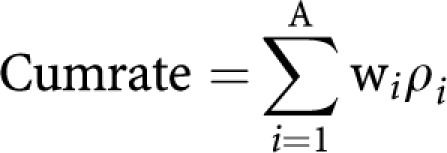
 where the summation is until age-band A, *w*_*i*_ is the width of the *i*th age band in years and *ρ*_*i*_ is the age-specific annual incidence rate in the ith age-band. This method has two advantages: first as a form of directly standardised incidence rate, comparisons between populations are immediately possible; and second it can be interpreted intuitively as an approximation to the cumulative risk an individual has of developing cancer up to a defined age, provided there are no other competing risks. However, the cumulative rate is far from ideal. A defined upper age limit needs to be chosen and can have a substantial impact on the result. For instance the cumulative rate to age 85 is for many cancers double the cumulative rate to age 75. If the upper age is set too low, say at age 75, then differences in cancer incidence or mortality between long-lived populations may be missed. Further, if the upper age is set too high, the intuitive interpretation as a risk of getting cancer is misleading because competing risks have not been taken into account and many individuals will die from an unrelated cause before reaching age 85.

*Cumulative risk* The cumulative rate can be converted into a true cumulative risk (Day, 1987) using the formula: 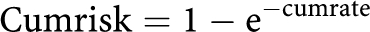 and, to a very close approximation, if the cumulative rate to a particular age is ‘1 in *x*’, then the cumulative risk to the same age will be ‘1 in (*x*+1/2)’. However, the correspondence between cumulative risk and cumulative rate is only valid if an individual is only able to have at most one event, which is clearly not the case in terms of incidence data for all cancers routinely presented by cancer registries. Although the cumulative risk does not give an estimate of the risk of developing cancer over a lifetime, it has been used as an approximation of this when the truncated upper age band is chosen as an age close to the average life expectancy of the population. However, neither the cumulative rate nor the cumulative risk take other competing risks into account, and hence tend to overestimate the probability of developing cancer over a lifetime, and indeed up to a particular age.

*‘Current probability’ method* A realistic estimate of the lifetime risk of getting cancer can be obtained by estimating the number of cancers that would arise during the lifetime of a hypothetical birth cohort. This was done by Goldberg *et al* in 1956 to estimate ‘the probability of developing cancer’ using a current life-table and calculating the number of cases that would occur within each age band (on the basis of the person-years at risk, from the life table, and the current age-specific incidence rate). This approach was termed ‘current probability’ by Esteve *et al* (1994). It takes competing risks into account and is not truncated at an arbitrary upper age; thus giving an estimate of lifetime risk. When truncated at the same age as a cumulative risk estimate, the ‘current probability’ value obtained is lower because it has allowed for the competing risks (Esteve *et al*, 1994; and Tables 1 and 2 below). However, comparisons of such lifetime risks between populations may not reflect differences in cancer incidence because the construction of the life table uses current all-causes mortality rates, which may differ between populations. Sasieni and Adams (1999) proposed using standard sex-specific life tables in an attempt to overcome this issue.

When the ‘current probability’ method is used on data containing only first primaries for all individuals, it provides an excellent estimate of lifetime risk (referred to here as the ‘gold standard’). However, when it is run on routine incidence data, two implicit assumptions are made, neither of which are likely to be exactly true. One is that the incidence rates are based on a denominator of individuals who have never had cancer before; the other is that the numerator only counts first cancers. Without such assumptions one is calculating a cumulative lifetime rate rather than lifetime risk. The issue of the numerator can be serious. Given the multiple primaries in routinely published incidence data, the ‘current probability’ method is actually estimating the average number of primary tumours per person, rather than the probability of getting cancer, and hence tends to overestimate the lifetime risk of getting cancer for all tumours.

*Devcan—the SEER analytical program adjusting the denominator in the current probability method* This package – available at http://surveillance.cancer.gov/devcan/ – uses a method that differs from the current probability method only in the way it deals with data in 5-year age bands. However, the authors of Devcan are also interested in the residual lifetime risk from a certain age and hence need to calculate the number of people who will be alive and cancer free at that age. This has been addressed by statisticians working for the US National Cancer Institute (Wun *et al*, 1998; Fay *et al*, 2003) building on the earlier work of Goldberg *et al* (1956). Devcan assumes that data with only the first primary tumour per individual are available. When estimating the lifetime risk of getting any cancer (i.e., at any site) using routinely published registry data, Devcan makes no adjustment for cancers at different sites in the same individual. Even with access to a registry database there is an issue of how far back the registry goes and how much immigration there is into the registry. With many registries an individual could have an earlier cancer that is unknown to the registry.

*The proposed new method – the adjusted for multiple primaries method* The issue of multiple primary tumours being recorded in the same person has been recognised previously, notably by the National Cancer Institute (Feuer *et al*, 1993) that used incidence data that only contained the first primary breast cancer diagnosis to calculate the lifetime risk of developing breast cancer, but has not been addressed (as far as we are aware) with respect to the risk of any cancer. Here we present a correction to address the serious issue of multiple primaries within routinely published incidence data: the adjusted for multiple primaries or AMP method.

Registries, such as the Scotland Cancer Registry, that are able to present data for only first occurrences offer an opportunity to assess the value of this correction, and the new method is illustrated in comparison with most of the methods described above to allow differences in the results of each approach to be examined. Additional analysis is undertaken using the new method on aggregated data in 5-year age groups, rather than on age in individual years, because this is the way routine incidence data are generally reported by cancer registries.

## Patients and methods

### Methods

The new formula is described with particular reference to estimation of lifetime risks of developing any cancer (as opposed to cancer at a single anatomical site), which is when the discrepancy due to registering multiple tumours is greatest.

Consider a multi-state model with the following states:

- Alive and never had cancer [0]
- Alive with/after cancer [C]
- Dead from cancer [D]
- Dead from something other than cancer [X]

Let *λ*_AB_(*t*) denote the hazard from state A to state B at age *t*. For simplicity, we often write *λ*_AB_ as a short hand for *λ*_AB_(*t*). Assume that *λ*_0D_=0 (i.e., one cannot die of cancer if one has never had cancer). Let *λ*_CC_(*t*) denote the hazard of getting a new diagnosis of cancer (at age t) in someone who has had cancer previously.

Let *λ*_C_ denote the observed cancer incidence rate – at age *t* – among those alive (whether or not they have cancer already). This can be written as a weighted average of the incidence rates of new cancers among those with and without cancer: 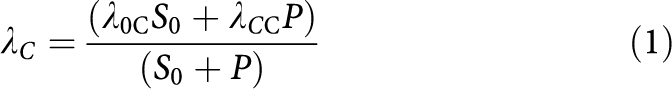 where *S*_0_ is an shorthand for 
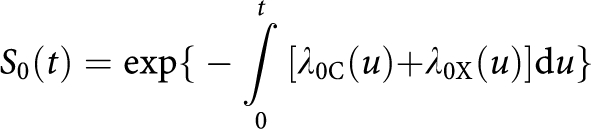
 the probability of being alive and cancer-free at age *t*, and *P* (a shorthand for *P*(*t*)) is the probability of having been diagnosed with cancer and still being alive at age *t*.

Similarly, let *λ*_X_ denote the observable mortality rate for causes other than cancer in the whole population. Again this is a weighted average: *λ*_*X*_=(*λ*_0*X*_*S*_0_+*λ*_*cX*_*P*)/(*S*_0_+*P*).

Now the lifetime risk of getting (a first) cancer is 
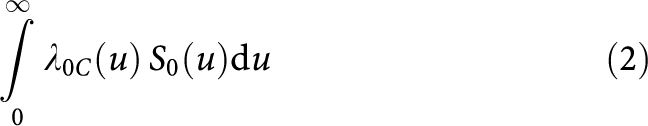
 but we cannot directly estimate *λ*_0C_(*u*) and *S*_0_(*u*) from routinely published cancer registry data. Instead, we suggest estimating lifetime risk by 
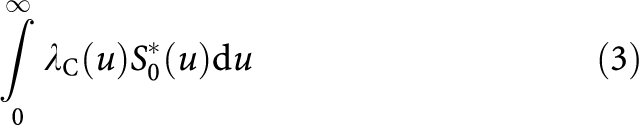
 where 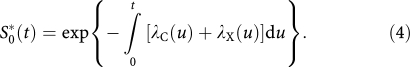


If, for all ages (using the shorthand form previously referred to), *λ*_0*X*_=*λ*_*cX*_ (i.e., the mortality from causes other than cancer is the same in those who have never had cancer and those who have had cancer) and *λ*_0C_=*λ*_CC_ (i.e., the incidence of cancer is the same in those who have never had cancer previously and those who have had cancer previously), then *λ*_0*X*_=*λ*_*X*_ and *λ*_0C_=*λ*_C_ and the formula used for estimation would be exactly equal to the formula used to define lifetime risk.

More generally, note that using (1), 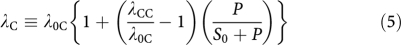 and similarly 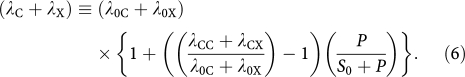


Note that *P*(*t*)/{*P*(*t*)+*S*_0_(*t*)} is the proportion of the population (alive at age *t*) with a previous diagnosis of cancer.

Thus the approximation is good when either the ratios *λ*_CC_/*λ*_0C_ and (*λ*_CC_+*λ*_C*X*_)/(*λ*_0C_+*λ*_0*X*_) are both close to unity, or when *P* is small in comparison with *S*_0_. When considering ‘all cancers’, it is reasonable to assume that *λ*_CC_/*λ*_C_ is close to unity: it was 0.99 when considering cancers other than at the original site in Denmark (Storm *et al*, 1985). However, for certain cancers *λ*_C*X*_ may be considerably greater than λ_0*X*_ due to a common risk factor such as smoking. Even when *λ*_C*X*_ is much greater than *λ*_0*X*_, the approximation may be good if the survival from the particular cancer is poor so that *P* is always small in comparison with *S*_0._ Lung cancer is an example for which the relative risk of a second primary in survivors is considerably greater than one, but the approximation is good because there are extremely few survivors of lung cancer.

#### Practical estimation using data in age bands

The following numbers are all assumed to be sex-specific. In age group *i* let:

*M*_*i*_ denote the annual number of deaths (all-cause mortality);

*D*_*i*_ denote the annual number of cancer deaths (cancer mortality);

*R*_*i*_ denote the annual number of (registered) cancer cases;

*N*_*i*_ denote the size of the mid-year population.

As shown in Appendix I, we can estimate the lifetime risk by 
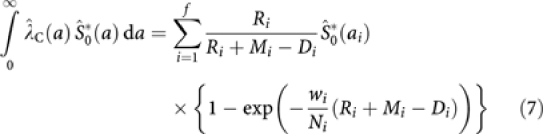
 where the i'th interval is from *a*_*i*_, to *a*_*i*+1_, *w*_*i*_=(*a*_*i*+1_−*a*_*i*_) and 
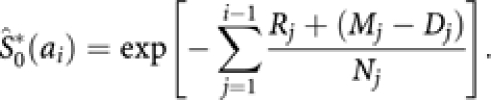


### Patients and populations

All-cause mortality and population data for Scotland for 2001–2005 were obtained from GRO Scotland. Cancer incidence and mortality data for 2001–2005 were supplied by the Scottish Cancer Registry. These were provided by sex and single year age bands for all cancers (excluding non-melanoma skin cancer (NMSC) for incidence, but including NMSC for mortality data; as is the usual practice by registries). Cancer incidence data were provided for (a) all primary registrations; and (b) only those cases where the diagnosis was the first tumour for that patient (i.e., only first primaries were included) for all cancers excluding NMSC. Data were also provided for breast cancer in women, prostate cancer in men and colorectal and lung cancers for both sexes, with and without multiple within site primaries. These sites were chosen to explore whether the correction for multiple primaries within site-specific cancers is necessary. For breast, lung and colorectal cancers, within-site multiple primaries are expected; in contrast, for prostate cancer, multiple primaries are not usual. Both the calculation of the usual current probability and the proposed adjusted current probability estimates are available in a bespoke Excel spreadsheet, which is available for download from both the National Cancer Intelligence Network (www.ncin.org.uk) and the Cancer Research UK websites (http://info.cancerresearchuk.org/cancerstats/).

## Results

Tables 1a and b show the results for males and females, respectively, obtained from the same input data, for all cancers (excluding NMSC) using six methods: cumulative rate, cumulative risk, ‘current probability’ (using all primaries), ‘gold standard’ (current probability run only on first primaries in single-year age bands), and the new ‘adjusted for multiple primaries’ method, run on both individual year of age data and on data aggregated in 5-year age groups. When restricted to the risk from birth to age 64 (i.e., under 65), the different methods produce very similar results. For risk calculated to higher age limits, the differences between the methods become more apparent. The cumulative rates up to the age of 84 for all cancers are 70% for males and 51% for females, with corresponding cumulative risks of 50% and 40%, respectively. These estimates are greater than the corresponding risks of developing cancer.

All of the methods, which give estimates of lifetime risk result in lower estimates than the cumulative rate or cumulative risk. The ‘gold standard’ estimates (‘current probability’ method on first primaries only) are 40% for males and 38% for females. In contrast, when the ‘current probability’ method is applied to all registered primaries, the estimates of the lifetime risk of cancer are 44% for males and 43% for females. Using the AMP method described in this paper on all primaries, the estimated lifetime risk of developing cancer is 39% for males and 38% for females for both the individual age and 5-year age group data; both of which are very similar to the gold standard estimates.

Comparison of results using routine incidence data for breast, lung, colorectal, and prostate cancers, with the ‘current probability’ method applied only to first primaries at a given site, showed smaller differences between the estimates (Table 2). For breast cancer, the AMP method using 5-year age bands overestimated the risk by only 0.3% compared with an overestimate of 0.8% using the ‘current probability’ method on all primaries. In contrast, for prostate cancer, the AMP method underestimated the risk, whereas the ‘current probability’ method gave an identical estimate to the gold standard (using only first primaries). For colorectal and lung cancers, there were almost no differences between any of the estimates.

## Discussion

Reliable estimates of lifetime risk can be difficult to obtain from routinely published incidence data. In this paper, we have described a new method for estimating lifetime risk that makes assumptions that are reasonable. The new method corrects for the inclusion of multiple primary cancers in the incidence rates published by cancer registries. It therefore provides a more accurate view of lifetime risk as opposed to the more commonly used measures based on mean number of events. The differences between the cumulative risk to age t, the ‘current probability’ estimate, and the new AMP estimate are shown by comparison of formulae in Appendix II (as suggested by a referee). Within the integral, the three quantities multiply the cancer rate by the following: the probability of being cancer free; the probability of being alive; and the probability of being alive and cancer-free, respectively.

Formulae are provided both for when the data are available in 1-year intervals and for when they are only available in 5-year age bands, as is often the case for those outside of cancer registries. The approximation using aggregated data (in 5-year age bands) is seen to be excellent. Fay (1994) discusses better approximations to be used when the data are only available in 5-year intervals.

The new method only requires one set of data in addition to the data required for the ‘current probability’ method; viz., data on cancer deaths for the specific site or group of sites. The four data sets required are therefore the population numbers, all-cause mortality, site/group specific cancer incidence and site/group specific cancer mortality.

The strengths of the new method are that it takes the competing risk of death into account and corrects for the fact that cancer registries publish data on all primary tumours that arise in each individual. Similar to the ‘current probability’ method, an arbitrary upper age limit is not required (in contrast to the cumulative risk method). Importantly, unlike other methods, the new method does not overstate lifetime risk for all malignant neoplasms: its estimated lifetime risk of developing cancer was 39% for males and 38% for females, very similar to estimates obtained using only first primaries (compared with 44 and 43% using the ‘current probability’ method).

The new method does make some assumptions, which, although unlikely to hold exactly, are reasonable for this sort of calculation. It assumes that the non-cancer mortality rates are the same in individuals without cancer as they are in the general population; and that the risk of (a new) cancer is the same in individuals who have never previously had cancer as they are in the general population. The multiple effects of smoking and social deprivation on both the incidence rates of several cancers and the mortality rates of non-cancer causes are examples of why these assumptions do not hold perfectly.

Nevertheless, the correction based on these assumptions would seem to provide a better estimate of lifetime risk than the one based on the usual ‘current probability’ method. It should also be emphasised that (like the ‘current probability’ method) the lifetime risk obtained from this calculation is an artificial construct and may not accurately reflect the actual lifetime risk from birth for any individual.

Breast cancer is a common cancer with reasonably good survival and women with a previous breast cancer have a higher incidence of breast cancer (subsequently) than the general population. Thus the assumption that the incidence is the same in those without breast cancer and in the general population is at best a rough approximation. Despite this, the new method overestimates the risk of breast cancer by only 0.3%, compared with an overestimate of 0.8% using the usual ‘current probability’ method.

Lung cancer is a further example where the assumptions are unlikely to hold exactly. In this case it is because common risk factors mean that death from other causes is more likely in those that have lung cancer than in those that do not. The empirical results show, however, that the results for lung cancer are excellent as would be expected, because unfortunately there are so few long-term lung cancer survivors in the population. For example, in England and Wales, fewer than 30% of lung cancer patients are alive 1 year after diagnosis, fewer than 10% are alive 5 years after diagnosis and only about 5% are alive 10 years after diagnosis (Cancer Research UK, 2011).

In contrast, the adjustment results in an underestimation of the lifetime risk of prostate cancer: the new method underestimates the risk by 0.3%. This is caused by an overcorrection as registration of more than one prostate cancer in an individual is very rare. This is the only site considered in this paper where the adjusted method performs less well than the usual ‘current probability’ method, when both are compared against the lifetime risk calculated using first primaries only. Indeed, we would recommend using the ‘current probability’ method (with no adjustment) for individual cancer sites (such as cervix uteri, corpus uteri, or prostate) where multiple within-site primaries are extremely rare and survival is reasonable.

It should be noted that the adjustment made by the AMP method is to reduce the ‘current probability’ estimate by a factor that is at most equal to the lifetime risk as estimated by that method and in practice the adjustment will be much less than this. Thus if a cancer has a lifetime risk of 3.0% by the ‘current probability’ method, the AMP method will give an estimate of between 2.9% (i.e., 97% of 3%) and 3.0%. Prostate cancer and ‘all uterine cancers’ are the only sites for which we anticipate that the AMP method will be appreciably worse that the ‘current probability’ method.

It should be noted that what we call the gold standard is based on cross-sectional estimates rather than cohort estimates of risk. A change in risk affecting a broad age-range over a relatively short period of time will be applied spuriously in the lifetime risk. The most common cause of such a phenomenon would be the introduction of some form of screening. With the introduction of PSA testing in the USA, the rates of prostate cancer rose sharply increasing by ∼50% in men aged 65+ between 1989 and 1992 (Stanford *et al*, 1999). By 1995 the rates in men aged 65–74 had fallen to just about 20% greater than the 1989 level, whereas in men aged 75+ the rates were substantially lower than they had been in 1989. Consequently the lifetime risk calculated using cross-sectional data in 1992 would have been substantially higher than that experience by any cohort.

## Summary

In reporting lifetime risk, it is important not to overstate the risk. To claim that any more than two out of five people will get cancer, based on the data from 2001 to 2005 for Scotland analysed here, is hard to justify as it does not take into account the chances that some of those cancers will be in the same rather than in different individuals. This is certainly not clear if one uses the cumulative rate or cumulative risk; nor is it clear if one uses the ‘current probability’ method to estimate lifetime risk, which results in a risk of 44% for males and 43% for females. Put another way, the difference between the ‘current probability’ and the new adjusted method can be roughly interpreted as the lifetime risk of getting two or more (independent) primaries. Thus, around 5% of people (1 in 20) get two or more primary diagnoses of cancer. It is important to note that changes in all-cause mortality rates over time may lead to an increase in the lifetime risk of cancer despite falling incidence rates. This increase is a consequence of longevity.

In conclusion, this paper offers an adjustment to the ‘current probability’ method, which enables a more accurate estimation of the lifetime risk of cancer to be calculated using commonly available 5-year age group data. Using the new method on all primaries for aggregated 5-year age group data for the UK for 2001-2005 data gives an estimate of the lifetime risk of developing any malignant neoplasm excluding NMSC in the UK of 40% for males and 37% for females.

## Figures and Tables

**Table 1:** Estimates of risk of developing any malignant neoplasm excluding NMSC, by calculation method; Scotland, 2001–2005: (a) males; (b) females

	**Age group**			
**Method**	**0–64 (%)**	**0–74 (%)**	**0–84 (%)**	**All ages (%)**
*(a) Males*				
Cumulative rate	15	36	70	
Cumulative risk	14	30	50	
‘Current probability’	13	27	40	44.4
AMP method	12	25	36	39.2
‘Gold standard’ – ‘current probability’ using first primaries^a^	12	25	36	40.0
AMP method using 5-year data	12	25	36	39.1^b^
				
*(b) Females*				
Cumulative rate	17	31	51	
Cumulative risk	15	27	40	
‘Current probability’	16	27	38	42.9
AMP method	15	25	34	37.7
‘Gold standard’ – ‘current probability’ using first primaries^a^	14	24	34	38.3
AMP method using 5-year data	15	24	34	37.6^c^

Abbreviations: AMP=adjusted for multiple primaries; NMSC=non-melanoma skin cancer.

a The incidence data used only includes the first primary diagnosis of any malignant neoplasm (excluding NMSC) for each patient.

b When a final age band of 85+ is used instead of 90+, the estimate is 39.2%.

c When a final age band of 85+ is used instead of 90+, there is no change in the lifetime risk estimate.

**Table 2:** Estimates of risk of developing cancer, by site, by sex; Scotland, 2001–2005

	**‘Gold standard’ – ‘current probability’**	**AMP method**	**‘Current probability’**	**Cumulative risk**	**Cumulative risk**
	**Lifetime**	**Lifetime**	**Lifetime**	**0–74 years**	**0–84 years**
	**First primaries only (%)**	**5-year data** **(%)^a^**	**(%)**	**(%)**	**(%)**
All malignant neoplasms (M)	40.0	39.2	44.4	30.5	50.3
All malignant neoplasms (F)	38.3	37.6	42.9	26.6	40.0
Breast cancer	10.8	11.1	11.6	9.2	12.5
Colorectal (M)	6.1	6.3	6.4	4.9	9.8
Colorectal (F)	5.0	5.1	5.1	3.0	6.0
Prostate	8.7	8.4	8.7	6.7	13.3
Lung (M)	8.7	8.8	8.8	6.9	13.4
Lung (F)	6.5	6.5	6.5	4.5	8.1

Abbreviations: AMP=adjusted for multiple primaries; F=female; M=male.

a Calculated using a last age band of 85+.

## APPENDICES

### Derivation of discrete data formulae

Let _C.*i*_=*R*_*i*_/*N*_*i*_ and _*X.i*_=(*M*_*i*_−*D*_*i*_)/*N*_*i*_. In contrast with the ‘current probability’ method, which only removes individuals from the cohort when they die, to obtain the number alive and cancer free, we also remove individuals as soon as they are diagnosed with cancer. To avoid double counting in estimation of the ‘removal rate’ we must therefore subtract the number of cancer deaths, *D*_*i*_, from the number of all deaths, *M*_*i*_, before adding the number of new cancers, *R*_*i*_. Thus

and more generally for an age a in the i'th age band

for a ∈(*a*_*i*_, *a*_*i*+1_).

In each age band we need to calculate the integral of  over the age band where the hazards (but not the survival function) are constant within the age band. For a 1-year wide age band from *i* to *i*+1 this integral is:

In practice, mortality and incidence are tabulated only up to an open-ended final age band (*f*,∞) (e.g., 85+). For the final age band the upper limit of the integral is infinity and as *exp*(–∞)=0, the integral for the final age band evaluates to the following:

More generally, because published data are typically supplied in 5-year age bands, writing *w*_i_ for the width of the *i*th age group (with *w*_f_=∞), we have

### Comparative formulae

Note that the gold standard is equivalent to the lifetime risk as defined in (2).
